# Physiological Action of Carbonic Acid

**Published:** 1870-12

**Authors:** 


### Miscellaneous.

### Physiological Action of Carbonic Acid.

Dr. Levin publishes (*Archives de Physiologie*) the following results of an investigation into the physiological action of carbonic acid: 1. When respired in the pure state or mixed with a certain proportion of air, it does not excite any convulsive action. 2. After absorption, it acts directly on the muscular fibres of the heart, modifying their chemico-physical and physiological properties, and destroying their contractibility. 3. It has no action on the blood-globules, nor on the blood-vessels. 4. It 'stupefies' the brain and the medulla oblongata: the stupefaction of the brain manifesting itself by suspension of its functions—of intelligence, sensibility and voluntary movement; that of the medulla oblongata, by arrest, succeeding impairment, of the respiration and circulation. 5. The reflex function of the spinal cord and the functions of the nerves are unaffected by this gas, and the contractibility of the muscles is likewise uninjured. 6. By the suspension of the functions of the brain and of the medulla oblongata, a condition of death-like sleep is produced, which can be removed only if it has existed for a certain limited period, varying with the species and age of the animal; and oxygen is the only substance that is capable of awakening the brain and the medulla oblongata from this death-like sleep. 7. If, by a proper mixture of carbonic acid and air, death is gradually produced (for example in about half an hour), the temperature of the body diminishes nearly two degrees centigrade, and diabetes supervenes. Sugar is found in large quantity in the blood and viscera; and in the rabbit the urine yields nearly 10 grammes of sugar to the litre. 8. Oxygen and carbonic acid produce contrary effects. The former excites the cardiac contractions, reddens the blood globules, gives life to the brain cells, stimulates the medulla oblongata, and acts peculiarly as a nourishing and vivifying gas; the latter, on the other hand, is a true poison, it is a gas that kills by destroying the physiological properties of heart, brain and medulla oblongata, and it is necessary that it should be continually eliminated. Dr. Leven's experiments were made on rabbits, cats and guinea-pigs; to whom the gas was administered either pure or mixed with definite proportions of atmospheric air, and either by inhalation during ordinary respiration or by introduction into the trachea through an artificial opening."—*Jour. Anat. and Phys.*, and *Amer. Jour. Med. Sciences.*

